# Corpus Callosum Size on Magnetic Resonance Imaging and Its Association With Developmental Delays in Children: A Retrospective Case-Control Study

**DOI:** 10.7759/cureus.99089

**Published:** 2025-12-13

**Authors:** Manik Mahajan, Vikrant Gupta, Smarth Nathyal, Sitikantha Banerjee

**Affiliations:** 1 Radiology, Government Medical College, Jammu, Jammu, IND; 2 Community Medicine, All India Institute of Medical Sciences, Vijaypur, Jammu, IND

**Keywords:** corpus callosum, developmental delay, hypoplasia, magnetic resonance imaging, white matter

## Abstract

Introduction

Developmental delay (DD) encompasses a heterogeneous group of conditions that present with a delay in development or an abnormal pattern of developmental progression. The corpus callosum (CC) is the largest white matter structure in the human brain and connects the cerebral hemispheres. The association between DD and CC thickness is poorly documented.

Objectives

The main objective of this study is to compare the thickness of various regions of the CC in children with and without DD, using magnetic resonance imaging (MRI).

Material and methods

This retrospective case-control study included 70 children aged two to six years (35 children each with DD and normal development). The thickness of the CC was measured on mid-sagittal T1-weighted images according to Witelson’s method. Associations between the mean sizes of the different regions of the CC in the case and comparison groups were assessed.

Results

Delayed speech was the most common presentation of DD, followed by delayed fine motor skills. A significant difference (p < 0.005) was observed between the mean thickness of the CC subdivisions in the case and comparison groups. The difference was most pronounced in the regions of the splenium, anterior body, and genu, with effect sizes of 1.59, 1.33, and 1.15, respectively (Cohen’s d values). Significant differences were also observed between the thickness of the genu and splenium in participants who predominantly displayed language delay (p = 0.013).

Conclusion

Corpus callosal thinning is associated with various DDs in the pediatric age group, and the presence of a thin CC on MRI should trigger evaluation for DD.

## Introduction

The corpus callosum (CC) is the largest area of white matter fiber in the brain and provides communication between the two cerebral hemispheres [1]. It constitutes the primary supratentorial cerebral commissure and integrates several cognitive functions [2]. The primary function of the CC is to transmit and integrate information generated by the two hemispheres [3].

Developmental delay (DD) comprises a heterogeneous group of conditions that begin in early life and present as a delay in development or an abnormal pattern of developmental progression. The term DD is defined as a significant delay (more than two standard deviations below the mean) in one or more developmental domains [4]. It is presumed that approximately 5%-10% of pediatric patients presenting in outpatient departments present with DD at various centers [5].

Thinning or hypoplasia of the CC may occur due to a number of causes, which can be divided into primary and secondary groups. The primary causes include leukoencephalopathies and inherited or acquired metabolic disorders. Secondary causes of CC thinning include hypoxic ischemic insult, viral infections such as HIV, hydrocephalus, or hypomyelinating/demyelinating conditions [6]. Certain neurodevelopmental disorders (e.g., dyslexia, attention deficit hyperactivity disorder, developmental language disorder, schizophrenia, Down syndrome) are also associated with corpus callosal alterations [7].

For evaluating DD in children, magnetic resonance imaging (MRI) is the imaging modality of choice. Around 60% of cases with DD present with abnormal findings on brain MRI [8]. The thickness of various parts of the CC can be reliably assessed using MRI. A brain MRI also reveals any previous injuries or other specific developmental abnormalities of the brain. Currently, only limited data are available regarding CC thickness in children with DD; this dearth is largely attributable to challenges involving technical barriers and the difficulty of securing cooperation from young children. This study aims to compare CC thickness in children with and without DD using mid-sagittal MRI imaging.

## Materials and methods

Study design

This retrospective case-control study was conducted in the post-graduate Department of Radio-Diagnosis and Imaging at a tertiary care center in North India. The study was approved by the Institutional Ethics Committee of Government Medical College, Jammu (IEC/GMCJ/2025/2092; dated February 11, 2025). The study procedure complied with the ethical principles outlined in the Declaration of Helsinki. Informed verbal consent to participate in the study, approved by the local ethics committee of the institute, was obtained from the parents/guardians of the participants, and human ethics guidelines were adhered to before performing the MRI study.

Study population

The study population comprised 70 children, ranging in age from two to six years, who had previously undergone MRI of the brain. The study group included 35 children diagnosed with DD on clinical examination by a pediatric neurologist, using the Denver Developmental Screening Test where applicable. The control group consisted of 35 age-matched children with normal development who had undergone MRI for other indications (e.g., headache, loss of consciousness, seizures, trauma).

Exclusion criteria

Patients with suspected metabolic disorders on MRI, patients who had undergone neurosurgery, those with lesions involving the CC or who displayed complete or partial agenesis of the CC, preterm children, uncooperative children, and scans with motion artifacts were excluded.

MRI acquisition and analysis

Imaging was performed in both groups with a 1.5T MRI (Siemens Somatom; Siemens Healthineers AG, Erlangen, Germany). Routine sequences (axial T1, T2, and FLAIR; sagittal T1 and T2; axial DWI and SWI) were used for imaging. Imaging parameters included slice thickness of 3-4 mm, matrix 256 × 256, TR/TE: T2 (4000/80), T1 (370/10), and FLAIR (8000/90), and FOV: 180-240 cm.

The thickness of the CC was measured on a mid-sagittal T1-weighted image according to Witelson’s method, which divides the CC into seven parts: rostrum, genu, rostral body, anterior midbody, posterior midbody, isthmus, and splenium [9,10]. The measurements were taken by two radiologists, each with at least five years’ experience in neuroradiology, after random allocation of image subsets of patients. Both radiologists were blinded to the clinical status of the child (case or control); however, neither inter-rater nor intra-rater reliability was assessed. IBM SPSS Statistics for Windows, Version 26 (Released 2018; IBM Corp., Armonk, NY, USA) was used for data analysis. The difference in the mean sizes of the various regions of the CC among the case and control groups was assessed using Student's t-test, and the effect was analyzed using Cohen’s d test. Statistical significance was set at p < 0.05.

## Results

In our study, the mean age of the children was 3.98 (±1.44) years. Among the study population, 40 children were male, with a M:F ratio of 1.33. Delayed speech (n = 27) was the most common DD in our study, followed by motor delay (n = 24).

Table 1 shows the mean thickness of the various regions of the CC in the case and control groups.

**Table 1:** Comparison of diameter of different sub-divisions of corpus callosum (mm) among cases (n = 35) and controls (n = 35) *Statistically significant with 5% alpha error

Sub-division of corpus callosum	Size in mm mean (SD)		t-test value	p-value	Effect size (Cohen’s d)
	Case developmental delay present (n = 35)	Control developmental delay absent (n = 35)			
Rostrum	3.88 (1.02)	4.52 (0.67)	3.12	0.003*	0.74
Genu	7.03 (1.82)	9.00 (1.03)	5.58	<0.001*	1.33
Anterior Body	3.45 (1.23)	4.73 (0.99)	4.74	<0.001*	1.15
Mid Body	3.32 (1.39)	4.31 (1.04)	3.39	0.001*	0.81
Posterior Body	2.76 (1.13)	3.58 (0.91)	3.34	0.001*	0.79
Isthmus	3.12 (1.28)	3.93 (1.04)	2.88	0.005*	0.69
Splenium	6.31 (1.68)	8.73 (1.34)	6.67	<0.001*	1.59

**Figure 1:**
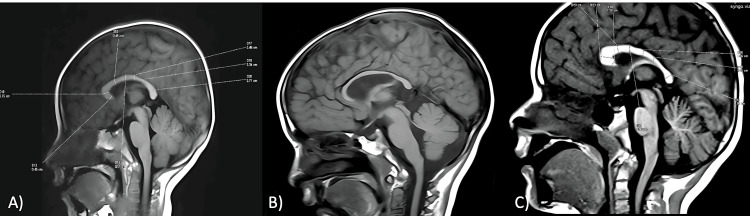
A) Measurement of various parts of the CC on sagittal MRI using Witelson’s method; B) Hypoplastic CC in a child with DD; C) Measurement of the CC in a child with DD CC, corpus callosum; MRI, magnetic resonance imaging; DD, developmental delay

**Figure 2:**
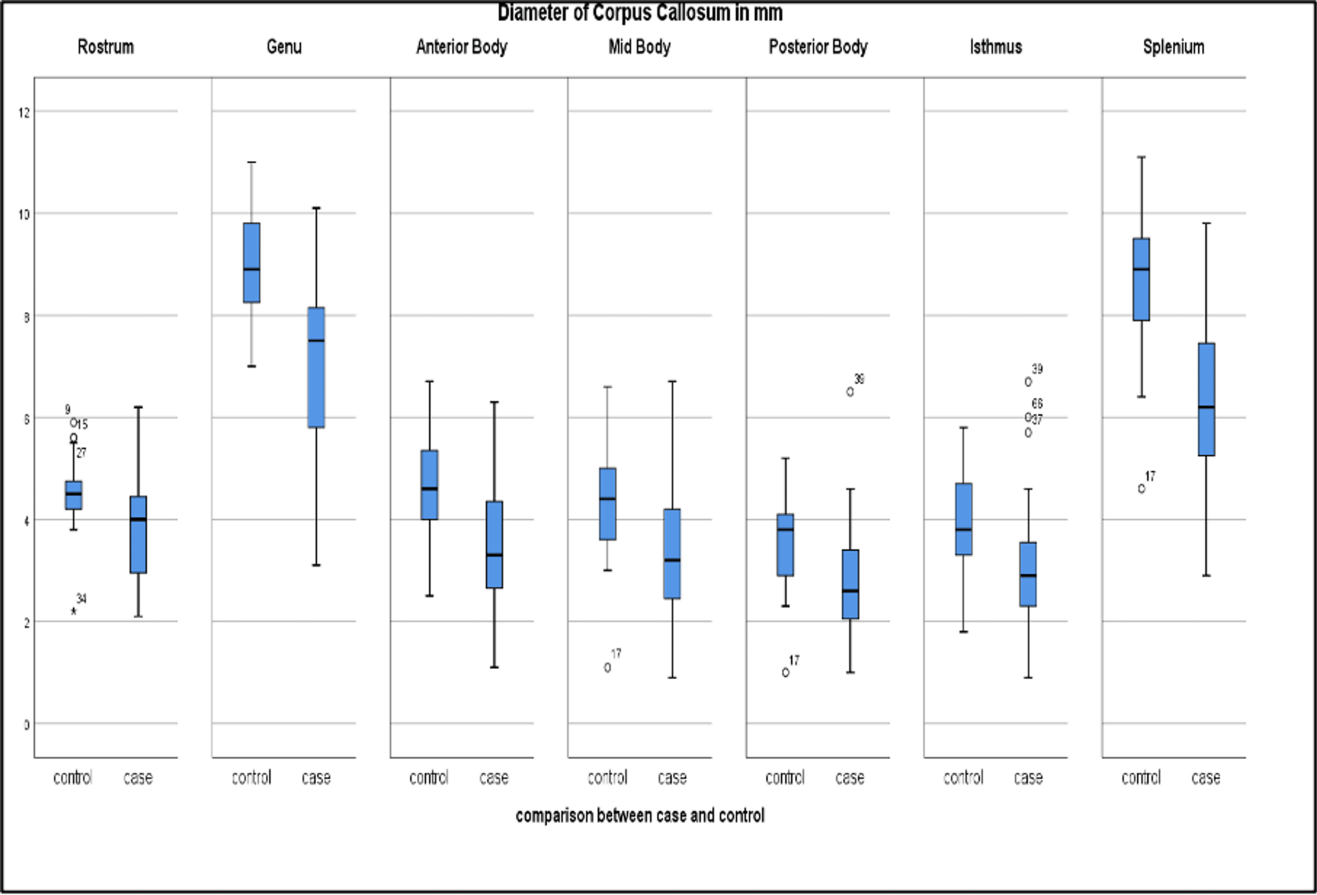
Box-plot diagram comparing diameters (mm) of different parts of the CC among patients with or without DD (n = 35 cases, n = 35 controls) CC, corpus callosum; DD, developmental delay

A significant difference (p-value < 0.005) was observed in the thickness of the CC on MRI between children with DD and normal controls (Table 1). Children with DD had smaller CC thicknesses across its various subdivisions (Figure 1). Further, this difference was most pronounced in the regions of the splenium, anterior body, and genu (Figure 2).

The largest effect was observed in the splenium (mean difference = 2.42 mm, Cohen’s d = 1.59), followed by the genu (mean difference = 1.97 mm, Cohen’s d = 1.33) and the anterior body (mean difference = 1.28 mm, Cohen’s d = 1.15). Predominantly, motor delay was observed in 22.9% of cases (n = 8), with the mean thickness of the genu and splenium being 5.06 mm and 6.07 mm, respectively. In patients with predominant language delay (n = 7; 20%), the mean thickness of the genu and splenium was 7.09 mm and 4.36 mm, respectively. A significant difference (p = 0.013) was observed between the thickness of the genu and splenium in patients with language impairment, whereas the difference in thickness of the genu and splenium in patients with motor delay was insignificant (p = 0.08).

## Discussion

In India, 1.5%-2.5% of children under two years of age are presumed to have DD [11,12]. These impairments impact not only the child and the family but society as a whole. The CC is the largest deposit of white matter fiber in the brain, and its thinning observed in DD patients may reflect disrupted myelination, reduced axonal density, or impaired interhemispheric connectivity, possibly influenced by genetic, metabolic, or environmental factors during early brain development. However, the association of a hypoplastic CC with DD is still not properly understood.

In this study, we compared the differences in thickness of various subdivisions of the CC in children with DD and in children with normal developmental milestones. A significant correlation (p-value < 0.005) was obtained between the mean thickness of the CC across its various subdivisions in both the case and control groups. The difference in thickness was most pronounced in the regions of the splenium, anterior body, and genu (Figure 2). In a similar study performed by Ravichandra et al. [13], a significant difference (p-value < 0.001) was observed in the mean thicknesses of various CC regions between the study and control groups. Chang et al. [14] performed a study to clarify the correlation between the condition of the CC and motor development in healthy, full-term infants; they observed a positive association between CC thickness and the development of rolling over. Smaller CCs have been observed in individuals with autism spectrum disorder, attention deficit hyperactivity disorder, and intellectual disability.

Stipdonk et al. [15] conducted a systematic review of school-aged preterm children on the relationship between language outcome and underlying brain structures and concluded that language skills and verbal fluency were strongly associated with CC volume. In our study, significant differences were observed in the thicknesses of the genu and splenium in patients with predominant language delay (p = 0.013), implying that the splenium has a predominant role in language development. In patients with predominant motor delay, the genu was more affected (mean thickness 5.06 mm) than the splenium (mean thickness 6.07 mm); however, the difference was insignificant (p = 0.08). In a similar study done by Northam et al. [16], significant white matter volume was observed in the splenium among individuals with language impairments.

Mandine et al. [17] performed a study to assess CC size in participants with DD. In their study, 73 children with DD were prospectively enrolled between September 2020 and September 2022, and CC size was evaluated using standard qualitative radiological assessments with an automatic quantitative tool. Hypoplastic CC was observed in 27.4% of cases with DD, reflecting the importance of the CC in neurological development.

Although a hypoplastic or thin CC is frequently associated with DD, a thickened CC may also present with DD. In a study by Lerman-Sagie et al. [18], nine cases with thickened CC were diagnosed on prenatal ultrasound. These patients had DD and seizures in early childhood. Many other syndromic conditions may be associated with enlargement of the CC, including neurofibromatosis type 1 and Cohen syndrome [19,20].

Our study had a few limitations. CC thinning or hypoplasia may also be seen in metabolic disorders, which are associated with DDs; those cases were not included in the study. Secondly, the sample size in our study was relatively small; a larger sample size or a multi-centric study is required to obtain more comprehensive results. Third, our study was a retrospective analysis. Also, inter-observer and intra-observer variability have not been assessed in the study. Lastly, potential selection bias may exist, as the control subjects were randomly selected from the pool of children undergoing MRI for non-DD-related reasons (e.g., trauma, seizure), and their neurodevelopmental status may not be completely typical.

## Conclusions

The CC is a major midline brain structure and is the most extensive neural pathway connecting the two homologous brain areas. Any pathology affecting the CC is associated with delayed development in children. Children with hypoplasia of the CC in our study exhibited gross motor or speech delay. Therefore, all children with a hypoplastic or thinned CC on imaging need clinical evaluation for DD, to allow early and accurate diagnosis and to initiate timely intervention.
